# Cytomorphometric Analysis of Buccal Exfoliated Cells in Geriatric and Pediatric Age Groups - A Cross-Sectional Study

**DOI:** 10.7759/cureus.39082

**Published:** 2023-05-16

**Authors:** Dharini S, Pratibha Ramani, Karthikeyan Ramalingam

**Affiliations:** 1 Oral Pathology and Microbiology, Saveetha Dental College and Hospitals, Saveetha Institute of Medical and Technical Sciences, Saveetha University, Chennai, IND

**Keywords:** cellular area, imagej, cytology, h and e, pap, nuclear cytoplasmic ratio, nuclear area, paediatrics, geriatrics, buccal smear

## Abstract

Background

We have to establish variations in cellular dimensions in buccal smears with respect to age. It can be used as a reference standard while dealing with age-related pathological abnormalities.

Aim

The study aims to compare the nuclear area (NA), cellular area (CA), and nucleus-to-cytoplasm ratio (N:C) of pediatric and geriatric age groups in smears obtained from clinically normal buccal mucosa.

Materials and methods

Buccal smears were collected from 60 subjects with age groups of <10 and >60 years. Cytological smears were prepared and fixed using alcohol. H&E and Papanicolaou's staining was performed as per manufacturer instructions. Cytomorphometric analysis was carried out using Image J software v.1.52 for CA, NA, and N:C. Statistical analysis using Student's t-test was performed using SPSS version 23.0 (IBM Inc, Armonk, New York).

Results

A significant difference (p<0.001) in NA and CA between the pediatric and geriatric age groups was noted. There was no significant difference in N:C among the study groups.

Conclusion

The present study provides baseline data of two different age groups that can be used for comparison of abnormal cells in suspicious clinical lesions.

## Introduction

The oral mucosa is a mirror that reflects an individual's health. It has efficient tissue homeostasis due to self-renewal properties [1]. The cell production in deeper layers is balanced by the loss of cells from the surface [2]. This state of equilibrium is controlled probably by feedback mechanisms that convey information from the process of desquamation on the superficial surface to the process of cell production in the deep layer. This kind of balance is known as a cybernetic system [3]. The unique self-renewal property is rhythmic in nature, and the time taken for a cell to divide and pass through the entire epithelium is called turnover rate or turnover time, which aids the thickness of the tissue to remain constant within relatively narrow limits. The turnover rate is 52-75 days in the skin, 4-14 days in the gut, 41-75 days in the gingiva, and 25 days in the cheek. In addition, Oral mucosa undergoes a scheme of differentiation that has various roles, such as stratification, cornification, cell to cell interaction contributing to its barrier function [4].

The dynamic properties of the oral mucosa are based on the underlying various regulating mechanisms. Any deviation from this leads to oral and systemic abnormalities. Apart from pathology, the multi-featured oral mucosa is influenced by various factors that revolve around one's physiological efficiency. Hence, the homeostatic imbalance results in the subsequent onset of illnesses inherent to the aging process [5]. The aging process starts right from birth, and our body undergoes various adaptations as it gets exposed to the environment and is subjected to lifestyle changes. The epithelial cells in the early decades may show differences when compared to that of other decades as it adapts to maintain their functions. Research over the past decades has neglected the gradual physiological alterations in cellular morphology and turnover with advancing age. Hence, this study is an attempt to establish physiological variations in oral epithelial cells with age that can be used as a reference standard in exfoliative cytology [6].

Our team has extensive knowledge and research experience that has translated into high-quality publications [7-10]. The aim and objective of the current study are to analyze cytomorphometrical features like cellular area (CA), nuclear area (NA), and nuclear-to-cytoplasmic ratio (N:C) of smears from the buccal mucosa and to compare the alterations between pediatric and geriatric subjects.

## Materials and methods

The current study was initiated after obtaining ethical clearance from the Institutional Review Board (IRB No. IHEC/SDC/OPATH-2102/22/661). Informed consent was designed according to the study, and approval was obtained from the ethical committee. The study considered those above 60 years of age as a geriatric category according to World Health Organization guidelines [11].

A total of 60 apparently healthy random individuals were selected from the outpatient department of the institution. Patients with clinically normal healthy mucosa were included in the study, and exclusion criteria included patients with a history of systemic illness, habits, oral potentially malignant disorders, oral infections, oral ulcerations, malignancy, and history of previous radio and chemotherapy as these conditions may influence the study results. Out of 60 study subjects, 30 were pediatric (<10 years), and 30 were geriatric (>60 years).

The patients were asked to rinse their oral cavities with water to clear any food debris. Exfoliated cells of buccal mucosa were collected using a wet wooden spatula by exerting gentle pressure. The scrapings were then smeared carefully on grease-free clean numbered new glass slides. The microscopic slides were immediately fixed in 95% ethyl alcohol in a clean Coplin jar for a minimum of half an hour. Papanicolaou staining was performed using the Rapid-pap TM Papanicolaou stain kit (Biolab Diagnostics, Mumbai, India) along with routine hematoxylin and eosin (H&E).

A cytomorphometric assessment was carried out. The Papanicolaou stained cells were examined under a 10x and 40x light microscope. Clearly defined cells without clumping and folding were considered for the study. For each slide, five different fields were chosen, out of which 10 epithelial cells were selected randomly per field. Five photomicrographs were taken for each patient at 10x. A total of 300 photomicrographs were assessed.

The cellular area and nuclear area of the exfoliated cells were measured using Image J software version 1.52 (Figure 2). The mean average of the cellular and nuclear area values obtained from ImageJ software was calculated and recorded for each subject. The nuclear-to-cytoplasmic ratio (N:C) ratio was measured from the nuclear and cellular area. The values were tabulated, and further statistical analysis was performed.

SPSS version 23.0 (IBM Inc, Armonk, New York) was used for statistical analysis. The difference in the nuclear area, cellular area, and N:C with age were analyzed using Student t-tests. A p-value less than 0.05 was considered statistically significant for all tests.

## Results

**Figure 1:**
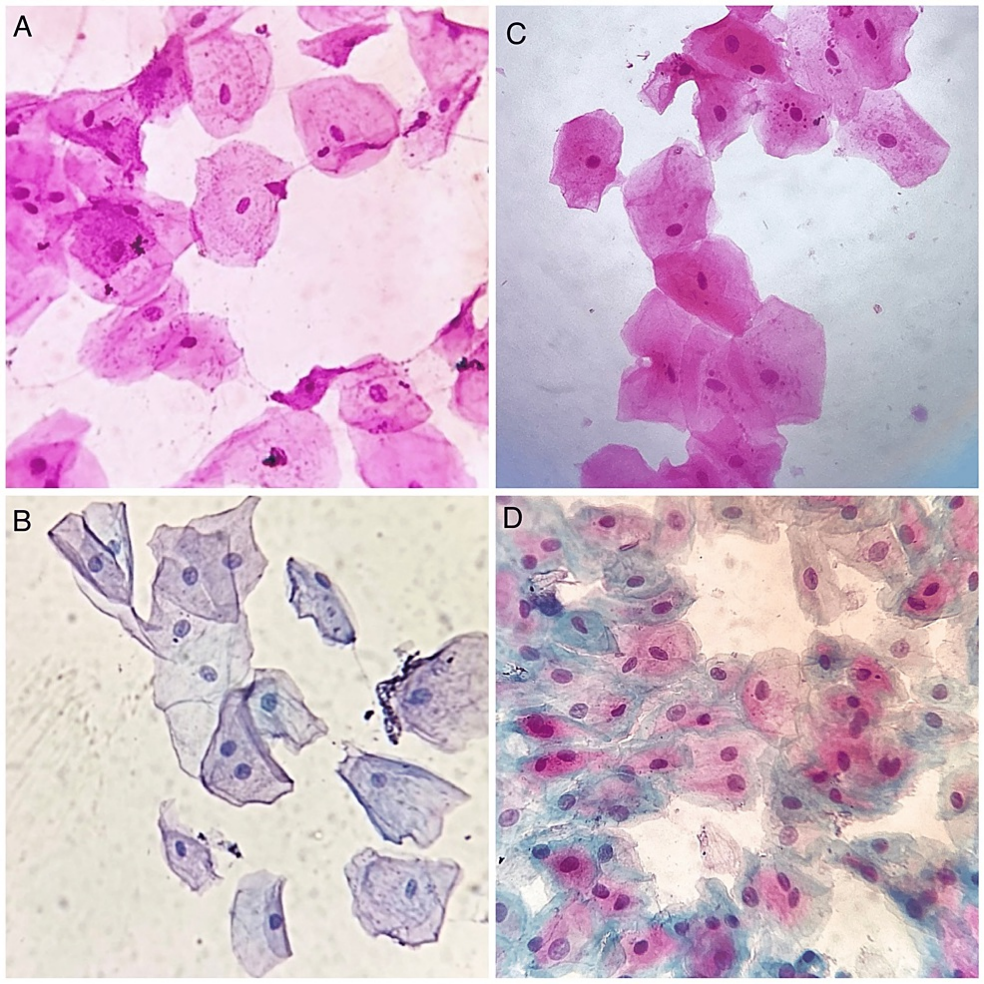
Photomicrographs of the study sample A) Photomicrographs showing exfoliated cells in geriatric patients (40x, H&E); B) Exfoliated cells in geriatric patients (40x, PAP); C) Exfoliated cells in pediatric patients (40x, H&E); D) Exfoliated cells in pediatric patients (40x, PAP)

This cross-sectional exfoliative cytology study was performed on buccal smears obtained from apparently normal patients of pediatric and geriatric age groups. A total of 60 samples were assessed in this study, with 30 samples in the pediatric group and 30 samples in the geriatric group. The mean age of pediatric patients was eight years, and the mean age of geriatric patients was 64 years. The pediatric group had 16 males and 14 females. The geriatric group had 19 males and 11 females. The buccal smears were stained with Hematoxylin and Eosin, and Papanicoloau staining (Figure 1).

**Figure 2:**
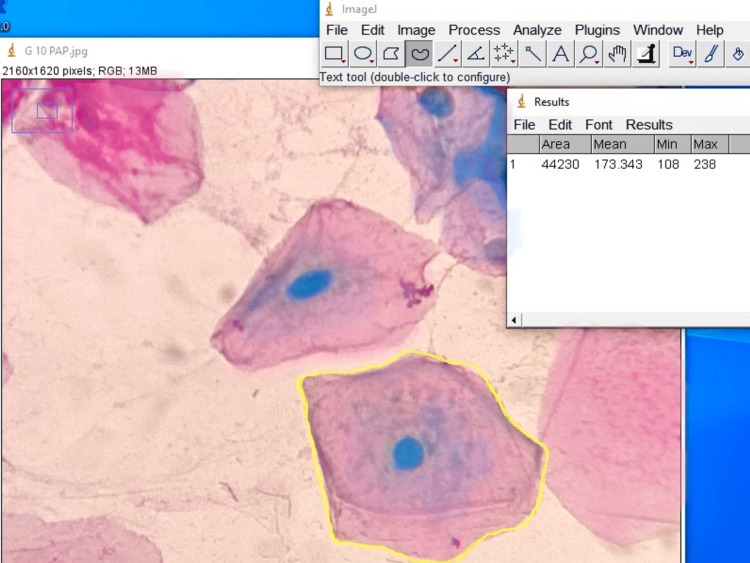
Image analysis Picture showing the cytomorphometric analysis using Image J Software

Cytomorphometric assessment was performed for the cellular area, nuclear area, and nuclear-to-cytoplasmic ratio using ImageJ software and tabulated for statistical analysis (Figure 2).

**Figure 3:**
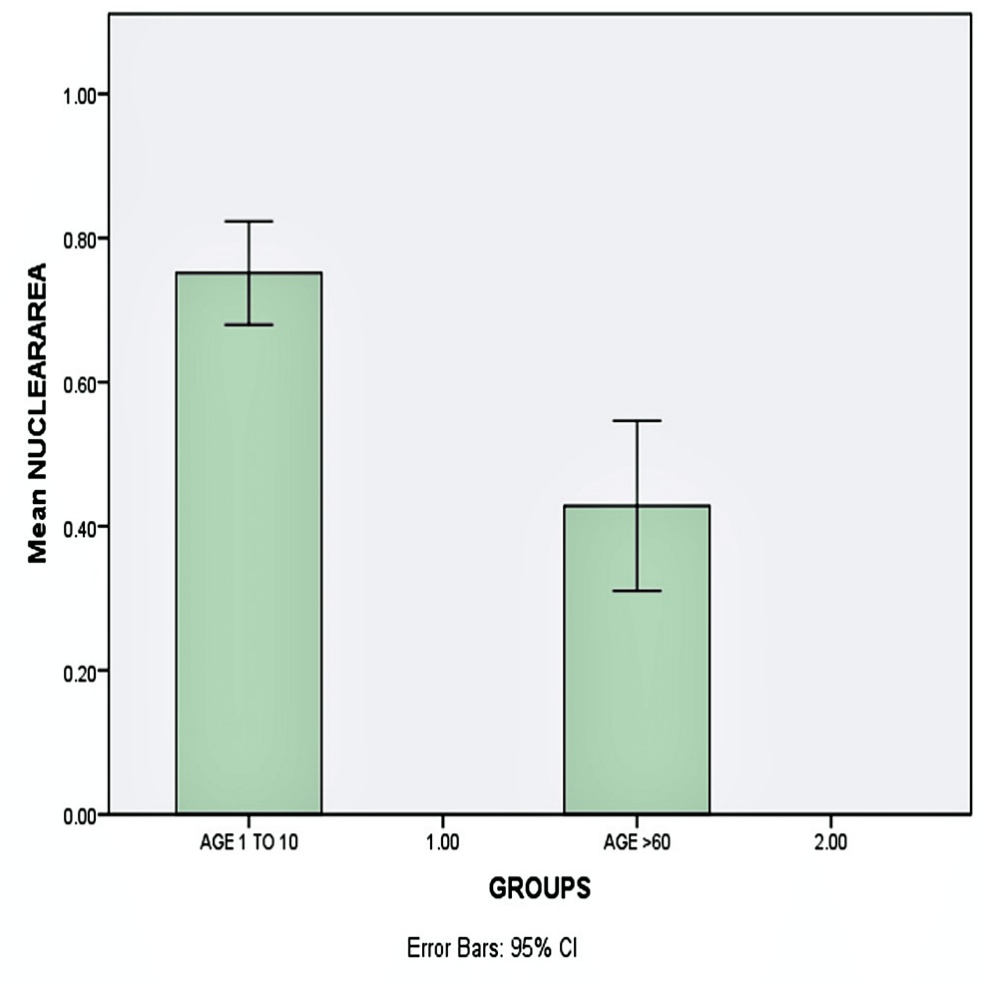
Graphical representation of the mean nuclear area Bar diagram showing the mean nuclear area of the pediatric population is greater than that of the geriatric population

The mean nuclear area was found to decrease from 0.751 in pediatric subjects to 0.428 in geriatric subjects (p-value <0.05), with a significant difference in nuclear area between pediatric and geriatric age groups (Figure 3). 

**Figure 4:**
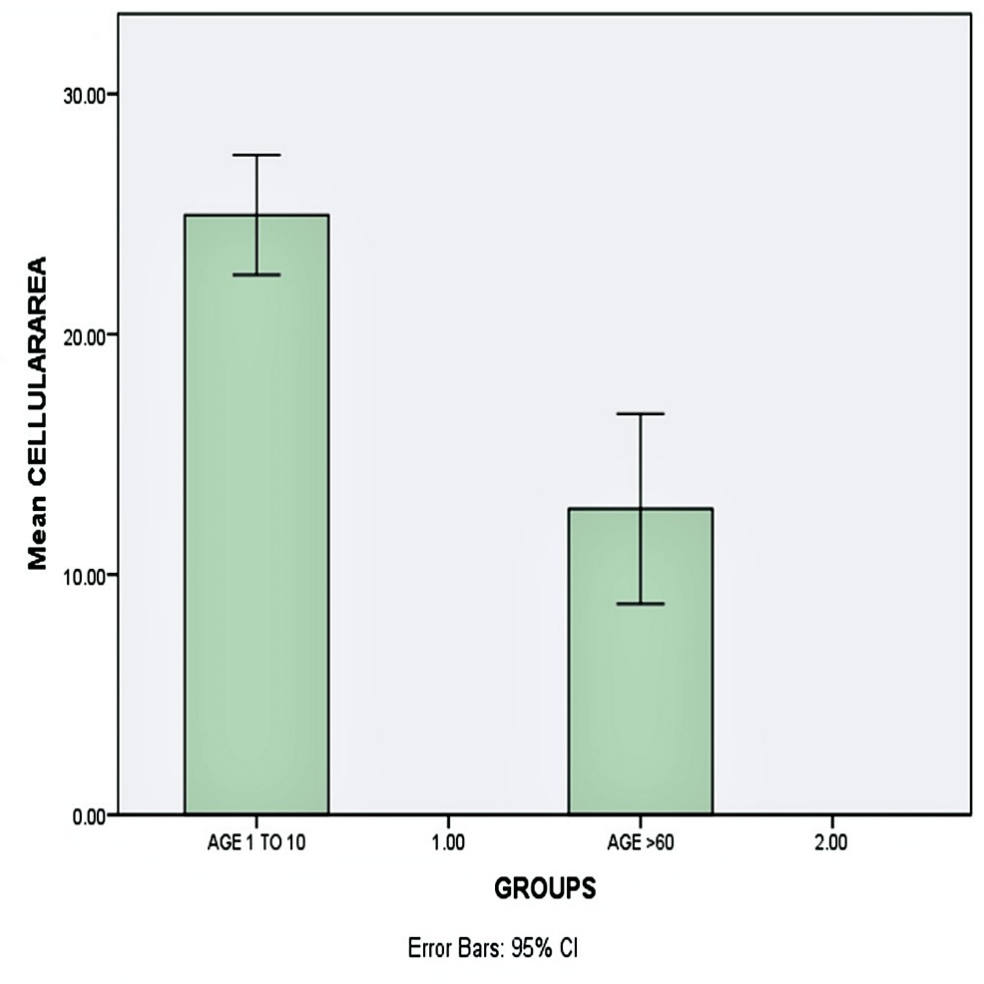
Graphical representation of the mean cellular area Bar diagram showing the mean cellular area of the pediatric population is greater than that of the geriatric population

The mean cellular area was found to be decreased by half, from 24.96 in pediatric subjects to 12.73 in geriatric subjects (p-value <0.05). There was a significant difference in cellular area between pediatric and geriatric age groups (Figure 4).

**Figure 5:**
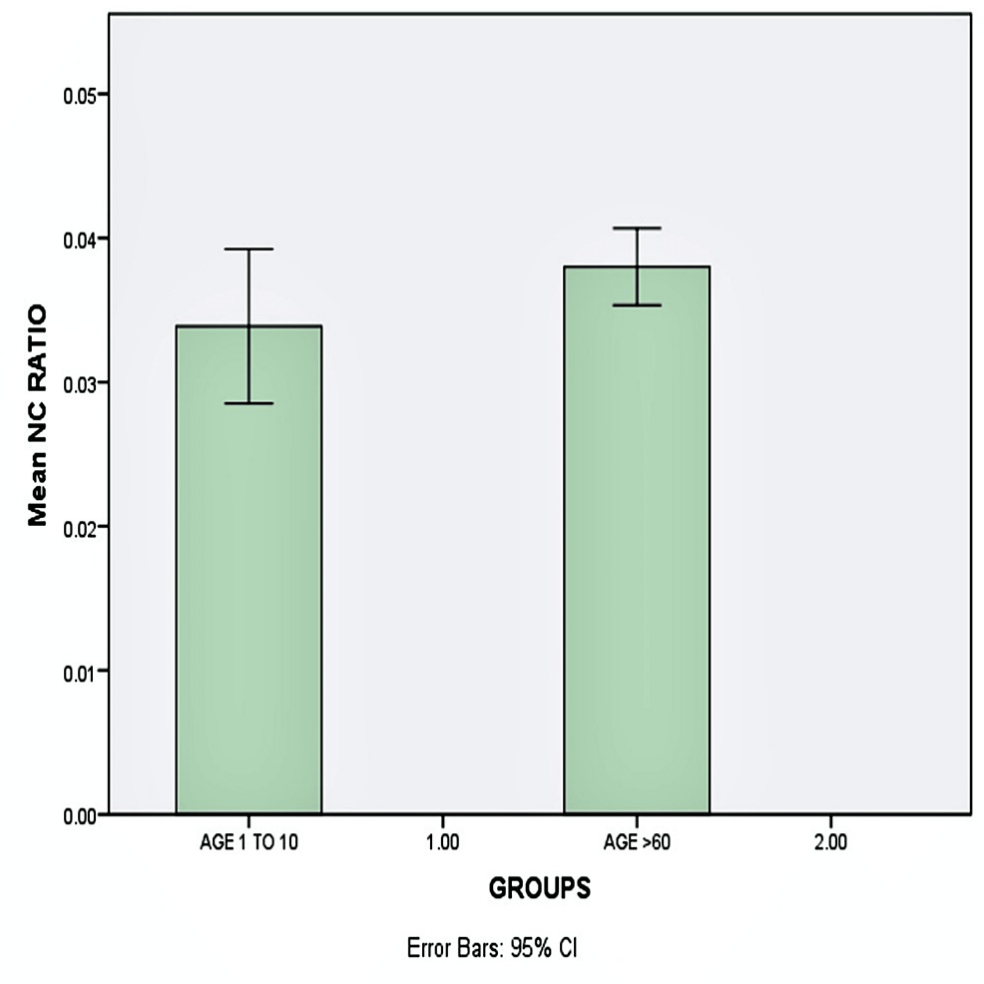
Graphical representation of the mean N:C ratio Bar diagram showing the mean nuclear-to-cytoplasmic ratio (N:C) between the pediatric and geriatric populations without any significant difference (p<0.05)

There was no significant difference in N:C among age groups, and the mean nuclear-to-cytoplasmic ratio was 0.039 in pediatric subjects and 0.038 in geriatric subjects (p<0.05) (Figure 5).

## Discussion

Analysis of cytological features involves various advantages, including simplicity and cost-effectiveness. It also reduces patients' anxiety as the diagnostic answers can be provided within one to two days. The cell features change in response to age as well as in a few abnormal conditions for which understanding the normal morphological characteristics of oral exfoliative cells is necessary for the effective identification of oral exfoliative cells. 

Very few studies have been conducted in the recent decade on buccal exfoliated cells [12]. In the present study, significant changes between cellular area (CA) and nuclear area (NA) and age groups (p=0.000) were observed. The nuclear-to-cytoplasmic ratio (N:C) between the two groups showed no significant changes. Pediatric patients may have larger cells due to their active metabolism and better self-renewal capacity, and vice versa in the case of geriatric patients. 

Cowpe et al. [13] compared normal oral squames between different age groups along with gender and site in order to provide baseline data and found that only nuclear size varied significantly with advancing age (F=2.61), and insignificant results were obtained for the cellular area (F=0.44). Insignificant results with regard to the cytoplasmic ratio in the previous study might be attributed due to failure in eliminating confounding factors that influence the cell's renewal properties. In addition, the age groups included in the previous study were from 21 to 85 years of age. Their results were consistent with the present study, but we also found significant variations in the cytoplasmic area.

Cytomorphometric alterations of oral exfoliated cells due to menstruation documented by Balan et al. [14] indicated hormonal change after puberty also has a major impact on the cells. Considering all these physiological changes that a body undergoes, the present study focused only on pediatric subjects less than 10 years of age in order to eliminate hormonal influences.

Priyadharshini et al. [15] studied exfoliated normal buccal mucosal cells of different age groups (10-20, 21-30, 31-40, 41-50, and more than 50) and found that there was a decrease in cellular and nuclear diameter (mean CD 51.97; mean ND 7.59) of pediatric age groups when compared to that of age groups above 50 years (mean CD 50.43; mean ND 7.18) and no variation was found in the N:C ratio. However, the evaluation of less than 10 years of age group was not given importance which makes the results less reliable to be used as a baseline value.

Substantial differences in oral exfoliated cells between smokers and non-smokers indicated environmental influence on cell turnover [16]. Significant differences between the 5-10 years age group and 15-35 age groups in terms of nuclear diameter and cellular diameter, which emphasized the impact of normal physiological alterations in the body on buccal exfoliated cells [1]. This may be attributed to the rise in estrogen and progesterone levels in their blood.

Studies by Oz et al. [17] and Ogura et al. [18] also reported significant variations in the cellular and nuclear area in type I and type II diabetes. This confirmed the influence of systemic conditions on the cell turnover rate. Hence excluding patients with systemic illness, habits, and other confounding factors in this study, we could provide reliable data to be used as a standard.

The future prospects include the use of specialized techniques such as liquid-based cytology and molecular analyses to enhance the specificity and sensitivity of newer stains and also to compare their efficacy with such modern techniques. Liquid-based cytology with cytomorphometric assessment, along with molecular and DNA analysis, could be included in future studies. [19]

Limitations

The limitations of the current study could be a small sample size and uneven gender distribution. The provided cytomorphometric data of pediatric and geriatric subjects in this present study aids as a baseline with which minor abnormalities with respect to any physiological and pathological changes can be compared. In addition, Oral exfoliative cytology is minimally invasive and is better accepted, especially in the pediatric and geriatric population, wherein patient compliance is already low. Cytomorphometric analysis using oral exfoliated cells can be used for periodic oral health examination and to monitor suspicious lesions wherein the cellular changes and nuclear changes can be assessed quickly.

## Conclusions

Understanding the normal physiological alterations in the morphology of oral exfoliated cells with age will prevent misinterpretation. The baseline data observed in this study could also be used for future research projects as well as in screening suspicious oral lesions. The present study showed significant changes in cellular and nuclear areas across geriatric and pediatric populations and concluded that the decrease in the cellular and nuclear area with age has an impact on the cell renewal property of the oral mucosa. Hence periodic evaluations of oral health can be done with oral exfoliative cytology smears, and subsequent cytomorphometric analysis will help in early identification and prompt management.

## References

[1] Donald PM, George R, Sriram G, Kavitha B, Sivapathasundharam B (2013). Hormonal changes in exfoliated normal buccal mucosal cells. J Cytol.

[2] Squier CA, Kremer MJ (2001). Biology of oral mucosa and esophagus. J Natl Cancer Inst Monogr.

[3] Hamilton AI, Blackwood HJ (1974). Cell renewal of oral mucosal epithelium of the rat. J Anat.

[4] Groeger S, Meyle J (2019). Oral mucosal epithelial cells. Front Immunol.

[5] Gregory AF, Antônio CN, Eneida MMC (2016). Effects of age on the frequency of micronuclei and degenerative nuclear abnormalities. Rev Bras Geriatr Gerontol.

[6] Matias AV, Cerentini A, Macarini LAB (2020). Segmentation, detection and classification of cell nuclei on oral cytology samples stained with papanicolaou. SN Comput Sci.

[7] Keerthana B, Priyadharshini R, Sinduja P (2021). Oral squamous cells and age estimation in exfoliative cytology with hematoxylin and eosin stain- a quantitative study. J Pharm Res Int.

[8] Krishna K, Gheena S, Santhosh MP (2021). Buccal mucosal changes in chronic alcoholics. Int J Dent Oral Sci.

[9] Govindarajan S, Veeraraghavan VP, Jayaraman S, Mony U, Sekar D, Patil S (2021). Use of toothbrush as a cost-effective noninvasive source of DNA for molecular Oral Oncology investigations during COVID pandemic. J Contemp Dent Pract.

[10] Anchana RS, Brundha MP (2020). Cytomorphologic analysis of wet fixed and air dried oral buccal smears rehydrated with normal saline: a comparative study. Int. J. Clin.

[11] Matsumoto MA, Castanho J, Kawakami RY, Ribeiro DA (2010). Cytogenetical damage in exfoliated oral mucosa cells in elderly people suffering denture stomatitis. Gerodontology.

[12] Sangamithra S, Arthanari A, Ramani P (2022). Cytomorphometric analysis of oral exfoliative cells for age estimation.. JPPW.

[13] Cowpe JG, Longmore RB, Green MW (1985). Quantitative exfoliative cytology of normal oral squames: an age, site and sex-related survey. J R Soc Med.

[14] Balan U, Gonsalves N, Jose M, Girish KL (2012). Symptomatic changes of oral mucosa during normal hormonal turnover in healthy young menstruating women. J Contemp Dent Pract.

[15] Ilayaraja V, Priyadharshini TK, Ganapathy N, Yamunadevi A (2018). Exfoliative cytology for age estimation: a correlative study in different age groups. Indian Acad Dent Spec Res.

[16] Godavarthy D, Rashmi N, Ahmed Mujib BR (2018). Tobacco-induced alterations in exfoliated oral epithelial cells: a comparative image analysis study. J Dr NTR Univ Health Sci.

[17] Oz ZS, Bektas S, Battal F, Atmaca H, Ermis B (2014). Nuclear morphometric and morphological analysis of exfoliated buccal and tongue dorsum cells in type-1 diabetic patients. J Cytol.

[18] Ogura Y, Akira F (2019). Exfoliative cytology of oral mucosa epithelium: cytochemical study and morphologic analysis of patients with type 2 diabetes.. Open J Stomatol.

[19] Sidhu SK, Ramalingam K, Goyal S, Poonia M, Rajawat GS, Sharma N (2018). Comparing the efficacy of Leishman-Giemsa cocktail stain, Giemsa stain, and Papanicolaou stain in potentially malignant oral lesions: a study on 540 cytological samples. J Cytol.

